# Vision and Control for UAVs: A Survey of General Methods and of Inexpensive Platforms for Infrastructure Inspection

**DOI:** 10.3390/s150714887

**Published:** 2015-06-25

**Authors:** Koppány Máthé, Lucian Buşoniu

**Affiliations:** Automation Department, Technical University of Cluj-Napoca, Memorandumului Street no. 28, 400114 Cluj-Napoca, Romania; E-Mail: lucian@busoniu.net

**Keywords:** unmanned aerial vehicle, control, planning, camera-based sensing, infrastructure inspection

## Abstract

Unmanned aerial vehicles (UAVs) have gained significant attention in recent years. Low-cost platforms using inexpensive sensor payloads have been shown to provide satisfactory flight and navigation capabilities. In this report, we survey vision and control methods that can be applied to low-cost UAVs, and we list some popular inexpensive platforms and application fields where they are useful. We also highlight the sensor suites used where this information is available. We overview, among others, feature detection and tracking, optical flow and visual servoing, low-level stabilization and high-level planning methods. We then list popular low-cost UAVs, selecting mainly quadrotors. We discuss applications, restricting our focus to the field of infrastructure inspection. Finally, as an example, we formulate two use-cases for railway inspection, a less explored application field, and illustrate the usage of the vision and control techniques reviewed by selecting appropriate ones to tackle these use-cases. To select vision methods, we run a thorough set of experimental evaluations.

## 1. Introduction

Unmanned vehicles, including UAVs, offer new perspectives for transportation and services. Although the legal requirements are still quite restrictive [1], UAV applications are becoming widespread, from military usage to civil applications, such as aerial imaging [2] or various inspection tasks [3,4].

We focus here on low-cost (under $1500), small-scale (diameter under 1 m) and lightweight (under 4 kg) UAVs that can be reliably used outdoors. Examples for UAVs that fit these criteria are the Parrot AR.Drone [5], the Arducopter platforms [6] or others presented in Section 4. While the specific limits on size, weight and cost are, of course, arbitrary to an extent, we are also motivated to select them by the railway inspection application we discuss in Section 6: since the UAVs are small they are unlikely to damage a train in the event of an unavoidable collision, and their low cost makes them easily replaceable. More generally, inexpensive UAVs are accessible to civilian users and commercially attractive for companies, making them more likely to become widespread. Among small-scale UAVs, higher-cost platforms, such as the AscTec, Draganflyer and MikroKopter products, offer improved flight stability [7,8] and advanced sensing units, such as laser rangefinders [9,10] or thermal infrared cameras [11,12]. While such platforms are sometimes also referred to as low-cost UAVs [12,13], we consider here significantly less expensive UAVs.

UAVs under $1500 use less expensive hardware, especially for sensing and processing units. They still possess basic navigation units, such as inertial measurement units (IMU) and possibly Global Positioning System (GPS) modules, but the measurement accuracy is usually reduced. Color cameras are used, which are useful only in daytime and do not provide depth and scale information for the captured environment. Nevertheless, the cameras are the richest data sources, so computer vision usually plays a central role in UAV automation. Building on vision, advanced sensing and control methods are used to compensate for the performance and capability limitations.

Therefore, in the first part of the paper, we survey general techniques for vision and control. We describe methods that work in any application, but are specifically motivated by infrastructure inspection, so we point out the connections of vision and control with this area. We begin by presenting at a high level existing vision methodologies and highlight those shown to be successful in UAV applications. The discussion is structured along several classes of techniques: feature detectors and descriptors to identify objects in the image, optical flow for motion detection and visual servoing and mapping techniques. The latter two techniques blur the line between vision and control, e.g., visual servoing tightly integrates visual feedback on the position relative to an object and the control actions taken to maintain the position. We continue by detailing UAV control methods, on two separate levels. For low-level stabilization and path following, we briefly introduce a simplified quadrotor model and methods for attitude and position control. We overview platform-independent high-level planning tasks and methods, focusing on methods that can integrate obstacle avoidance and other constraints.

Already for low-level control, and also for the remainder of our paper, we select quadrotors as our preferred class of UAVs. Among the two main types of UAVs, fixed-wing and rotorcrafts, rotorcrafts have the important capability of hovering. Subtypes are helicopters and multirotors, where multirotors are preferred for the robustness and modularity of the fuselage, being less subject to damage and easier to repair. Furthermore, quadrotors are the most widespread and least costly multirotors. Since we are interested in automating broadly-used UAVs, in this paper, we will focus on quadrotor platforms.

We start the second part of the paper by overviewing several low-cost quadrotor platforms. We then introduce the main focus of this second part, infrastructure inspection, and review UAV applications in this area. Selecting a less explored subfield, namely railway inspection, we develop two use-cases in this field to illustrate semi-autonomous short-range and fully-autonomous long-range inspection. We start with a detailed comparison of the performance of various feature detectors, based on real data from the use-cases. This also serves as a detailed illustration of the practical performance of many of the vision techniques we review. Based on the test results, we select appropriate vision algorithms. In addition, we select control methods based on our literature review, for each scenario, adapted to the facilities of the Parrot AR.Drone quadrotor.

In related work, a number of surveys address specific sensing, vision and control topics for UAVs. For example, the recent survey of Whitehead *et al.* [14] evaluates sensor types used for remote sensing applications. In the context of vision methods, [15] presents extensive datasets and benchmarks for optical flow techniques, and [16] discusses in detail edge detection methods. On the control side, [17] presents techniques for low-level stabilization and control, ranging from simple linear to more accurate nonlinear controllers, while [18] discusses high-level planning. Another survey [19] overviews planning methods from the perspective of uncertainties. The definitive book on robotic planning [20] also addresses low-level dynamics as constraints on planning. We do not intend to duplicate these efforts here. Instead, we provide an overall, high-level overview of both vision and control, focusing on methods relevant to recent low-cost quadrotors and infrastructure inspection applications. Indeed, we refer to these existing works, drawing on their comparisons between available methods and synthesizing their results, so in a sense, our paper is a meta-review of the area. Of course, due to the wide research fields discussed, the overview provided by us is not exhaustive. In particular, we do not include state estimation methods, used for example to infer unmeasurable variables from measured data or to perform sensor fusion. Our major goal is to help the practitioner in the areas of inspection with UAVs understand what methods are available and how they organize in a coherent landscape, so as to select an array of techniques for their specific application; and we provide the relevant references needed to implement the chosen techniques.

The next two sections present our survey on vision (Section 2) and control methods (Section 3). Then, Section 4 lists popular low-cost UAVs; Section 5 discusses common UAV monitoring and inspection applications; Section 6 evaluates vision techniques and selects control methods from those discussed for two illustrative use-cases; and Section 7 concludes our survey. Throughout, we pay special attention to sensor suites and present them whenever this information appears in the cited papers. Specifically, in Section 2.5, we discuss sensors used in vision-based applications. We highlight in Section 4 sensors found in low-cost platforms, and in Section 5.4, we present the sensor suites considered in infrastructure applications.

## 2. Vision: Camera-Based Sensing and Image Processing

Automated navigation of UAVs inherently requires sensing. Usually, ultrasonic sensors, color, thermal or infrared cameras or laser rangefinders are used to acquire information about the surrounding environment. From these sensor types, low-cost UAVs often possess color cameras. Information is then extracted using computer vision techniques: the acquired images are processed for stabilization, navigation and further information collection.

In UAV navigation, feature detectors and extractors are often used for object detection; optical flow techniques are used to distinguish motion in a scene; visual servoing is employed to translate image frame motion into UAV displacement; whereas 3D reconstruction methods are exploited for navigation and mapping. The literature on computer vision is rich (see surveys [15,21–23]), and instead of reproducing these efforts, here, we briefly introduce the aforementioned classes of vision techniques, highlighting popular methods and providing a list of relevant references for further reading. We also exemplify the use of specific methods in inspection applications. Later on, in Section 6, we will evaluate the performance of existing implementations of several vision methods on real data taken from our railway inspection use-cases.

An important remark regarding most vision methods is that they are well known as being difficult to rank by performance. Each method is suitable for specific types of environments and target objects. Evaluation methodologies, like the one presented by Rockett [24], exist, but the performance of vision methods remains subject to the fine-tuning of their parameters according to the problem at hand. Nevertheless, certain methods are preferred either for their robustness, flexibility in parameter selection or lower computational demands, as highlighted in the sequel.

### 2.1. Feature Detection and Description Methods

Feature detection and description algorithms are basic tools for object detection and tracking. These methods are used, for example, to extract UAV position and motion information. Methods differ from each other in the preprocessing used (grayscaling, blurring, masking), in the way the features are interpreted and selected, and in the mathematical operations used in the processing steps.

Features determine regions of interest in images, which are classified roughly as edges, corners and blobs. Detection methods are responsible for identifying them, whereas descriptors are used to match a feature in two images (e.g., images from a different perspective or subsequent frames from a video stream). Detectors in combination with descriptors and matching methods form complete tools for motion tracking. They can be also used for object detection given a reference image of an object, and in this context, additional tools, like the model fitting methods, can be considered. In what follows, we first discuss edge, corner and region detectors, then descriptor methods. We present then feature matching, homography-based detection and model fitting methods for object detection.

Edge detection is usually employed to identify lines and planes in images. Some of the classic methods are Canny, Sobel, Laplacian and Scharr edge detectors [25]. Several surveys exist that compare the performances of these and other algorithms [21,26,27]. A survey performed by Oskoei *et al.* [16] highlights the good performance of step edge models used for feature lookup and Gaussian filtering considered for further image processing. A classic example of such a method is the Canny edge detector. However, it is known to produce false edges for noisy images, and therefore, methods like the Haar wavelet transform [26] can be considered when the performance of the former is not satisfactory.

Corner and region detectors are mainly used for finding and tracking objects. The Harris–Stephens [28] and Shi–Tomasi [29] methods are often used. A recent study performed by Tulpan *et al.* [30] compares four corner detectors in their performance of identifying distant objects for sense-and-avoid applications. They compare the Harris–Stephens, smallest uni-value segment assimilating nucleus (SUSAN) [31], features from accelerated segment test (FAST) [32] and Shi–Tomasi methods on real video streams. Their results show that the Shi–Tomasi and Harris–Stephens methods outperform the others when looking at the execution time, while the Shi–Tomasi method has the best results concerning the detection range and the ratio of frames containing the detected target.

Feature descriptors are used for matching features in image pairs, either for detecting motion or for finding objects. Well-known methods are speeded up robust features (SURF) [33] and scale-invariant feature transform (SIFT) [34], whereas from recent years, we mention binary robust independent elementary (BRIEF) [35] and oriented FAST and rotated BRIEF (ORB) [36]. SIFT and SURF are older methods and are superseded by BRIEF and ORB in execution time while keeping comparable accuracy. On the other hand, ORB overcomes the limitations of BRIEF in processing rotations in images.

Given the feature descriptors, features can be matched in pairs of images. This can be achieved by simply comparing the descriptors from the two processed images and marking the closest descriptor pairs. This approach is called brute force feature matching. Other solutions consider search trees for comparing the descriptors. A popular matcher uses approximate nearest-neighbor (ANN) search [37] that offers a more efficient way to match features than the brute force approach. A well-known implementation of ANN is the Fast Library for ANN (FLANN) [38]. Using matched features, methods like the homography transform [39] determine the transformation of the image compared to the reference image, and from there, they infer displacement and rotation. A recent comparison of camera pose estimation algorithms using stereo cameras shows that the homography method provides an acceptable level of detection and is useful for applications with computational constraints [40].

Model fitting methods are often used in object detection. They categorize image features to find inliers and outliers according to a model (a curve or a shape described mathematically). A well-known example is the random sample consensus (RANSAC) method. A comprehensive performance evaluation of the RANSAC family is performed by Choi *et al.* [23], showing an accuracy and robustness improvement for maximum likelihood estimation SAC (MLESAC). RANSAC is used in several UAV applications, e.g., for wall plane detection [41] or for identifying the ground plane [42,43]. Although RANSAC methods aid the identification of objects having various shapes, they require high processing power and, thus, are less preferred in applications where computation is limited. Another classical model fitting method class uses the Hough transform; see surveys [44,45]. Hough transforms are most often used as straight line detectors and, thus, are preferred for linear structure detection. From the numerous variants [45], we point out the progressive probabilistic Hough transform for its faster computational performance and for the available implementation in OpenCV [46].

In UAV inspection applications, feature detectors are useful for detecting targets (buildings, objects) or references that have to be followed (like linear structures or the horizon). For linear structure detection, edge detectors can be combined, e.g., with line extractors. To achieve target detection, feature detectors can be coupled with descriptors, matchers and reference images. When combined with descriptors and matchers, feature detectors can also be used to track moving objects or to keep a reference position relative to a detected object.

### 2.2. Optical Flow Techniques

Optical flow is a family of techniques that focuses on determining motion from images. More precisely, optical flow can be defined as the apparent motion of feature points or patterns in a 2D image of the 3D environment [47]. Often, optical flow detection is performed, e.g., with an optical mouse sensor, as it works on a similar principle and is a popular, well-tested device [47]. A comprehensive analysis of existing optical flow techniques is performed by Baker *et al.* in [15]. As stated in their concluding remarks, at the time of their publication, the most promising optical flow detection approach was the one presented by Sun *et al.* [48]. In a more recent study by Sun *et al.* [49], they show, among others, the good performance of classic optical flow formulations.

Although providing useful navigation information, optical flow algorithms are usually time consuming. Chao *et al.* [47] remark for instance that usually, optical flow algorithms perform well with image sequences presenting much slower motions compared to UAV flights. These methods can nevertheless still be considered, for example, in near-hovering operation of UAVs.

### 2.3. Visual Servoing

Building on the techniques discussed before, visual servoing deals with controlling the camera (and, therefore, vehicle) motion using visual data. In the context of UAV navigation, visual servoing methods offer solutions for translating image frame motions into real-world displacement. Examples of applications of visual servoing in inspection are flight around traffic signals, following gas pipeline or scanning building façades.

In its classical formulation, visual servoing builds upon feature detectors. Novel approaches consider other parameters (mainly global histogram parameters) of the images as features and perform visual servoing using this information [50–52]. We concentrate next on feature-based visual servoing, which has two major directions: position-based visual servoing (PBVS) and image-based visual servoing (IBVS). PBVS finds a position estimate of the camera by calculating the pose of a known object in the image and uses this position to correct the motion path [53]. IBVS simply selects and tracks features in the images, using feature descriptors shown in Section 2.1, and corrects the position of the camera so as to keep the desired position of the features in the image frame [54]. Although simpler, IBVS may suffer from drift, and thus, it is often used only in combination with other navigation methods (e.g., GPS/IMU-based control) or as a fallback solution. The pros and cons are thus complementary for the two approaches: PBVS can ensure accurate positioning, but at high computational costs, while IBVS is faster, but may result in following undesired trajectories. Implementation examples and further discussions can be found, e.g., in papers [55,56].

### 2.4. 3D Reconstruction Methods: Mapping

Beyond finding the pose and the motion of the camera, simultaneous localization and mapping (SLAM) constructs a map of the environment while localizing the vehicle on this map. Localization then allows for autonomous navigation in unknown environments. Of course, SLAM is useful for mapping applications. In the case of camera-based SLAM, depth information, needed for finding distances and scaling factors of objects, can be collected by means of extra sensors or by using reference tags. For low-cost solutions, the latter technique is preferred, which calculates the distance from the size of known objects or tags, captured by the camera.

SLAM requires high processing performance, and its implementation is time consuming. A common implementation of SLAM is parallel tracking and mapping (PTAM). PTAM addresses the processing issue by executing UAV tracking (localization) and mapping in two parallel threads: the first thread tracks the camera motion (localization step), while the second one adds new information to the map (map refinement step) [42]. Despite parallel execution, PTAM remains a computationally-consuming algorithm, especially due to the map that requires more processing as it grows.

Yang *et al.* [42] consider PTAM to find an indoor landing site autonomously, using a single down-looking camera. They propose to obtain a constant computation time of PTAM by avoiding refinement of the entire map and performing it only locally, around the current position of the quadrotor. Similarly, Schauwecker *et al.* [43] consider refining maps only locally and clearing old frames in order to boost PTAM. They find that using down-looking cameras, the algorithm can have problems in tracking the quadrotor's motion in the case of yaw rotation (around the *z* axis). Using stereo cameras, they can correct for these errors in the case of slow rotations.

Although research in the field of computer vision is extensive, there remain several open challenges. Most of the vision algorithms are subject to fine-tuning and are limited in use to certain conditions, specifically due to illumination conditions, pattern types and the motion of captured objects. Another issue is the increased processing time of these methods, which needs to be further reduced for online applications on devices with limited processing power. Additionally, the lack of scaling and distance information in the case of 2D images leads to the need for further tools (sensors or techniques) for acquiring this information. Though the existing solutions are promising, further development is needed for having more robust techniques with wider applicability.

### 2.5. Sensors in Image Processing Applications

From the above listed works, several provide details on sensor platforms. We have found, e.g., that Tulpan *et al.* [30] use a Prosilica GC2450 5 MP monochrome camera for image processing, operating at 14 fps. Flores *et al.* [41] consider Kinect, a commercially available camera system, consisting of a low-cost RGB-D and infrared camera, providing 640 × 480 pixel RGB images and 320 × 240 pixel depth images, both at 30 fps. Yang *et al.* [42] work with a Firefly MV monochrome camera that provides 640 × 480 pixel images at 60 fps and a 90-degree viewing angle. Schauwecker *et al.* [43] consider a dual stereo-camera solution, mounting two pairs of 640 × 480 grayscale cameras on their UAV, one pair facing downwards, recording at 15 fps, and another facing ahead, recording at 30 fps.

One may conclude that, in most cases, images not larger than 0.3 MP are used for image processing, at frame rates up to 30 fps. This resolution is far lower than those offered by the currently available cameras, though it is preferred for keeping computation low (higher resolution images would require more processing time) and also for requiring only low-cost devices. Most research results, including the above ones, show that this resolution is good enough for proper detection. Furthermore, in terms of control, the acquisition frame rate of 30 fps is high enough, e.g., for controlling small-scale UAVs, and exceeds, in many cases, the processing rate that vision methods can offer.

## 3. Flight Control and Planning

Relying on information provided by sensing, effective control can help in overcoming the limitations of inexpensive sensors. Indeed, several works report good results using low-cost platforms with advanced controllers [57–59]. In general, the task of an unmanned aircraft is to safely navigate on a desired path and/or visit points of interest in order to perform certain missions. Control tasks behind these terms were grouped by Amelink [60] in the following levels of abstraction, from top to bottom: mission, navigation, aviation, flight control and flight. In this manner, he clearly classifies control problems and offers a modular approach to UAV control.

Instead of such a detailed decomposition of UAV control problems, we prefer to discuss control tasks at two levels: low-level flight control and high-level flight planning. With this grouping, the lower level covers control tasks that are often already implemented in UAVs: hovering ability and disturbance rejection, achieved mainly by attitude control, and trajectory following, the result of position control. By an abuse of terminology, we will refer to all of these tasks as UAV control in this section. On the other hand, high-level flight planning is then made responsible for mission and path planning, including obstacle avoidance. We will call these problems together UAV planning.

Many of the methods presented, especially the higher level planning techniques, are platform independent. Low-level control will be discussed from the perspective of quadrotors, the platform type we will use in our illustrative use-cases and also in our future work. Therefore, first, we introduce the dynamic model of a quadrotor, followed by detailing low-level control and high-level planning methods.

### 3.1. Quadrotor Dynamics

**Figure 1:**
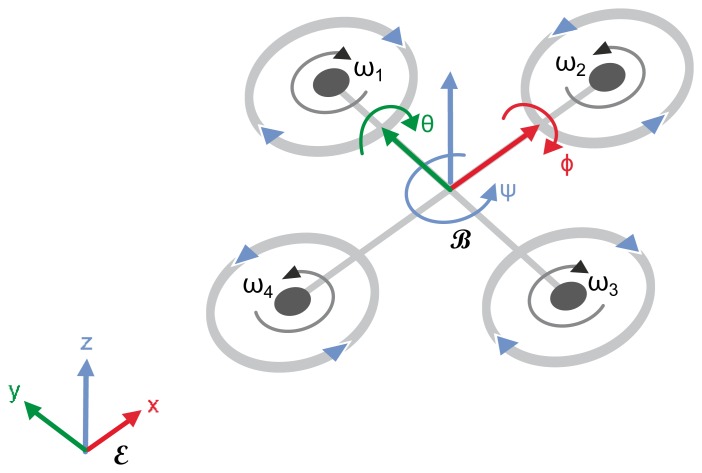
Quadrotor model.

A basic scheme of a quadrotor is shown in Figure 1, where ℰ denotes the Earth frame, also called the inertial frame; ℬ denotes the body frame, attached to the center of mass of the quadrotor; *x*, *y* and *z* mark the coordinates of the center of mass of the quadrotor, in the Earth frame ℰ; *φ, θ* and Ψ correspond to the conventional roll, pitch and yaw angles; and *ω_i_* marks the angular velocity of each rotor separately.

In the “plus” configuration, where the vehicle axes correspond to the *x* and *y* axes of frame ℬ, displacement along the *x* axis can be obtained by pitch rotation, which results from keeping *ω*_1_ = *ω*_3_ and setting *ω*_2_≠*ω*_4_. Similarly, flight on the *y* axis results from yaw rotation, i.e., *ω*_2_ = *ω*_4_ and *ω*_1_≠*ω*_3_. Hovering, lift and landing can be achieved by having velocities of the same magnitude on all propellers, whereas rotation around the *z* axis is the result of *ω*_2_=*ω*_4_ ≠ *ω*_2_=*ω*_3_. Then, control commands can be defined as:

(1) $$\begin{array}{l} U_{\text{coll}}=b(\omega_{1}^{2}+\omega_{2}^{2}+\omega_{3}^{2}+\omega_{4}^{2})\\ U_{\varphi }=b(\omega_{1}^{2}-\omega_{3}^{2})\\ U_{\theta }=b(\omega_{4}^{2}-\omega_{2}^{2})\\ U_{\psi }=d(\omega_{2}^{2}+\omega_{4}^{2}-\omega_{1}^{2}-\omega_{3}^{2}) \end{array}$$

where *U_coll_* denotes the collective input (responsible for vertical displacement), *U_φ_* the roll (*y* axis movement), *U_θ_* the pitch (*x* axis displacement) and *U_Ψ_* the yaw forces, *b* is the thrust coefficient and *d* is the drag coefficient. With these four inputs, the quadrotor can be operated simply in non-acrobatic flight mode (non-acrobatic flight maneuvers mean that the quadrotor's velocity is changed slowly and the vehicle is used to fly most of the time parallel to the Earth, up to some tilt being necessary for horizontal displacement). Note that all of these motions are with respect to frame ℬ, which then have to be transformed into Earth frame ℰ.

Now, the reaction to the control inputs, *i.e.*, the flight dynamics of the quadrotor, can be modeled using the Euler–Lagrange approach. A simplified form of the dynamics in near-hovering mode can be written as [8]:

(2) $$\begin{array}{lll} \ddot{x}=\theta g & \ddot{y}=-\varphi g & \ddot{z}=\frac{\Delta U_{\text{coll}}}{m}\\ \ddot{\varphi }=\frac{l}{I_{x}}U_{\varphi } & \ddot{\theta }=\frac{l}{I_{y}}U_{\theta } & \ddot{\psi }=\frac{1}{I_{z}}U_{\psi } \end{array}$$

Often, more general forms of this model are considered [61,62]. However, even those works build on the assumptions of near-hovering operation mode, low flight speeds and that the quadrotor can be modeled as a rigid body and has a symmetric structure. As these are realistic considerations for most low-cost small-sized quadrotors, controllers based on the principle of Equation (2) can be easily transferred from one platform to another, where only some parameters have to be adjusted to match the new vehicle.

### 3.2. Low-Level Flight Control

**Figure 2:**
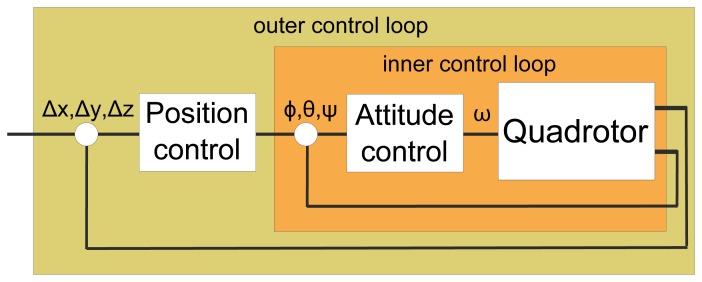
Nested low-level control loop: attitude and position control.

The main low-level control tasks are: achieving flight, stabilizing the UAV and following a flight path. These tasks are addressed by attitude and position control, which, in the case of quadrotors, are commonly coupled as a nested control loop, shown in Figure 2. Attitude control is then responsible for flight stabilization and tracking the desired heading, while position control serves for trajectory following.

Attitude control is often addressed by using proportional integral derivative (PID) controllers. Often, the PID controllers are set up by experimental tuning. They have the advantage of requiring no complex model of the system dynamics. Based on the results from papers [57,63], this control method, although simple, provides good results for the attitude control. However, the attitude controller is usually enhanced with robust features for obtaining improved stability [57,64].

Concerning position control, Raffo *et al.* [64], for example, propose to use an error-model based model-predictive control that simulates a virtual quadrotor following a desired trajectory. The role of their controller is to minimize the position error between a real and a virtual quadrotor. They compare this solution in a simulation to a backstepping approach. Both methods show robust tracking performance, though the former solution leads to smaller errors and smoother control.

Attitude and position control are often discussed together. Cui *et al.* [63] perform, for instance, trajectory tracking, using PID controllers for position control and several controller types for setting the attitude of the simulated vehicle. In simulations, they show that the PID controller provides the smallest tracking error and lowest settling time for attitude control. Salazar *et al.* [62] combine PID with robust sliding mode control (SMC) for attitude and position control in performing trajectory tracking. They conclude that, despite the good control performance of SMC, chattering of the control input may lead to quick wearing of the actuators, and thus, the use of SMC might be a less preferred solution.

Despite the good performance of current low-level stabilization systems and controllers, several further challenges remain to be addressed. Among these are the various types of uncertainties appearing in outdoor operation, the integration of saturation limits in the control schemes and the underactuated nature of the systems. In recent works, such challenges were addressed by more advanced control methods, such as adaptive controllers [8] or model-predictive control [65].

### 3.3. High-Level Flight Planning

The higher level problems of UAV automation relate mainly to defining and planning missions, as well as to planning flight paths that fulfil these missions. The goal is to make UAVs fly autonomously based on a mission plan and to make the flight paths feasible and optimal.

Path planning provides flight paths for the lower-level trajectory tracker. It results in optimization problems where certain costs (e.g., energy consumption, execution time) have to be minimized to find an optimal path. To achieve online execution, often, receding horizon techniques are considered. In the sequel, we detail the principle of receding horizon planning and present works that use it for UAV path planning. Afterwards, we discuss planning constraints that ensure flight path feasibility and methods to address them.

Exhaustive overviews of path planning methods can be found in [18–20,66]; see also the references therein. Here, we provide some reference works in the field of UAV path planning.

The idea of receding horizon planning is to reevaluate solutions while closing the control loop. In control, this approach is called model predictive control (MPC). At each call, MPC evaluates possible control sequences for a period of time called a horizon. Each sequence has an associated cost. After the simulation stops, MPC follows the control sequence with the best cost for a time period called the execution horizon. The algorithm is then repeated from the new state. MPC can cover nonlinear dynamics problems, too, such as UAV path planning and path following under uncertainties. However, especially in that case, MPC becomes time consuming, mainly due to “repeated” calculation of the cost values that usually requires simulating the model of the system.

For reducing computation, a common planning method used for UAV planning is the rapidly-exploring random tree (RRT) algorithm. RRT propagates random samples in the search space of control sequences, rapidly covering in this manner the possible solutions. Lin *et al.* [66] use RRT in a receding horizon fashion and couple it with Dubins curves in order to find feasible paths online, paths that avoid moving obstacles. Dubins curves are curves that connect two points while respecting constraints on the curvature. Although the RRT method cannot ensure global optimality [66], shows the practical success of the method both in simulations and in real flights.

Bellingham *et al.* [67] use MPC for path planning among obstacles. They work with linearized models and simplify the cost value calculation by using a cost estimator in order to reduce the execution time. They manage to obtain near-optimal solutions in around a minute [67]. More recent works report on using MPC for more complex situations, such as path planning for multi-UAV formation flights among obstacles [68] and with communication constraints [69]. However, the computational demand of these solutions is not transparent and is likely to exceed the processing performance of low-cost platforms.

An important challenge comes from the feasibility constraints considered in the planning. These integrate kinematic constraints (coming, e.g., from obstacles) and dynamic limitations (mainly due to the velocity and acceleration limits of the vehicle) [70]. Feasibility constraints can be treated in several ways. Commonly, they can be implemented as equality or inequality constraints in the optimizer [67,68,71] or can be simulated as curves [66,72,73]. For example, mixed-integer linear programming (MILP) is an optimizer that can integrate both linear and binary constraints. Bellingham *et al.* [67] use, for instance, MILP for solving path planning, while considering both the continuous constraints resulting from the dynamics of the vehicle and binary constraints coming from obstacle avoidance rules. In their RRT approach discussed before, Lin *et al.* [66] use Dubins curves for their planning method in order to cover the dynamic constraints of the vehicle.

Alternatively, kinematic constraints (e.g., obstacles) in particular can be addressed by means of control methods from computer vision; see Section 2. Navigation methods, such as visual servoing or control based on optical-flow, can be considered. Furthermore, to reduce the problem complexity, obstacle avoidance can be addressed with position control, where the avoidance maneuver represents a temporary deviation from the planned trajectory [74]. Similarly, dynamic constraints can be applied to adjust the flight path after it has been planned. Methods, such as the artificial potential field, can be used to smoothen the trajectory [75]. Such solutions are often less time consuming and may be preferred when computation power is limited. However, they do not offer performance guarantees and might be applicable only in certain scenarios, where waypoints are not mandatory to be reached, but deviations from the planned trajectory that are small in a certain sense are acceptable.

On top of flight planning sits mission planning, which covers the descriptive part of UAV automation. Mission planning includes all formal and informal requirements and specifications regarding usage of the vehicle. Furthermore, it is responsible for translating the missions into a well-defined sequence of subtasks that can be interpreted by path planners. Such subtasks are: take-off, fly to a coordinate, track an object, *etc*. Mission planning can be formalized, and the translation of missions into subtasks can be then performed automatically, based on a set of subtasks and rules. Looking at the literature, only sparse work has been done in this direction, mainly related to airspace integration efforts [1,76,77].

Several open challenges exist in UAV high-level planning. The computational demand of the advanced path planning methods is often high compared to the processing capabilities of low-cost UAVs, meaning that adaptations of these techniques are required in order to implement them onboard and online. Computational limitations also require approximate models, linearization or discretization of the system. Regarding the automation tasks, obstacle avoidance remains the most demanding problem, mainly due to the big variety of avoidance cases, which are hard to address all at once. On the other hand, mission planning challenges mainly relate to airspace integration issues, for which it is important to have a proper formalization of tasks in order to clearly define the expected and possible flight phases and events.

### 3.4. Flight Control and Planning Methods for Inspection Applications

Low-level stabilization is a basic flight requirement, and thus, attitude and position control are mandatory. However, the required flight planning techniques will vary. If one can assume obstacle-free flight when keeping a reference distance to the inspected target, visual servoing or preplanned trajectories can be considered. For online obstacle avoidance or for power consumption optimization online replanning, methods like the RRT should be considered. MPC-based techniques are able to explicitly deal with more complex constraints on the inspection (e.g., covering both flight time and obstacle avoidance) and are thus preferred in environments with more obstacles (e.g., urban roads, train stations).

## 4. Low-Cost Quadrotor Platforms

Having overviewed vision and control techniques, we move on to available low-cost platforms and UAV applications. The market of low-cost UAVs has exploded in the past two years. New brands and models of UAVs continuously appear, mainly quadrotors and helicopters, and several communities [78,79] and websites [80–82] focus on the evolution of these products. Instead of looking at the newest platforms resulting from startups and other research projects, we focus on popular brands. Working with a widespread platform usually ensures that the hardware is well tested and that the platform has long-term support, compared to more custom solutions. We therefore list several representative UAV platforms, available until April 2015, that also satisfy our criteria defined in Section 1.

We focus mainly on ready-to-fly (RTF) vehicles, having the advantage of less time needed for setup and calibration. However, RTF vehicles are usually limited in programmability and, therefore, are less customizable. This aspect does not limit the manually-teleoperated inspection applications, but is relevant for automated flights. Thus, we discuss with each presented platform the level of customization, as well. We also compare these platforms with a category of modular solutions that overcome these limitations. For each platform, we highlight the sensors used. In general, all of the quadrotors listed have onboard stabilization, some type of camera (embedded or as a separate device) and wireless connection for data transmission, and most of them include a GPS module, as well. Details are presented below.

Parrot released its popular AR.Drone 2.0 in 2012 citeweb:parrot. Edition 2.0 costs $400 and weighs 420 g. It has two onboard, built-in cameras, a bottom quarter video graphics array (QVGA) camera (320 × 240 px resolution) with 60 fps for ground speed measurement and an HD front camera (1280 × 720 px resolution) with 30 fps for video recording. The flight stabilization system consists of a set of three-axis gyroscope, accelerometer and magnetometer, enhanced with a pressure sensor that, together with the previous, makes the quadrotor able to withstand winds of up to 24 km/h [83]. A pair of ultrasonic sensors help in altitude keeping in close-to-terrain flights (up to 6 m). The onboard flight controller runs in Linux and is closed-source; however, using the Parrot SDK or other APIs, one may remotely access functionalities, such as: receive online camera image frames, navigation and other sensor data and issue high-level velocity commands on all axes, as well as take-off and land commands. These operations are limited to the range of the WiFi signal used for communication with the quadrotor. Various projects target extending the control range or allow for onboard programmability, though not as part of the official product. An official extension by Parrot is a GPS module that, for an additional $120, among others, allows for flight path scheduling by specifying waypoints, even outside the WiFi range. However, the quadrotor is not controllable outside the WiFi range, which limits it to short-range usage. Besides this popular model, Parrot released the Bebop drone at the end of 2014. For $500, among others, Bebop provides improved processing image capturing capabilities (14 Mpx image and 30 fps video recording at 1920 × 1080 px), has a built-in geo-location system (GNSS, including GPS and GLONASS) and an operation range of 250 m [84]. The additional Skycontroller, costing around $400, allows for extending the operation range to 2 km.

Similar products are the Xaircraft X650 Pro [85], having a SuperX onboard flight controller [86], and the Mikrokopter Quadrokopter XL [87], using the FlightCtrl ME flight controller [88]. The price of these UAVs is around $1000, and they weigh around 1000–1500 g. Based on the flight controller specifications, both controllers use for stabilization a set of sensors like those presented with the AR.Drone: pressure sensors, three-axis gyroscopes, magnetometers and accelerometers. A special feature of the controller of the Mikrokopter UAV is that its altitude sensing works up to 5000 m. Both UAVs are meant for mounting external cameras, where the X650 Pro flight controller has built-in functionalities for the camera gimbal stabilization and control. Theoretically, any recording device can be attached to these UAVs, up to the payload limit (around 1 kg for both UAVs).

DJI produces quadrotors for aerial imaging [2]. For the price of around $1000 and weights of about 1200 g, these vehicles are known to be stable. However, the flight controllers used with them allow only for path scheduling based on GPS waypoints, using a graphical interface. The publicly available specifications provide less technical information about the flight controllers [89]. Some of their proprietary flight controllers support the attachment of a GPS module. The newer platforms have proprietary cameras attached to the UAV through a two-axis gimbal, providing HD 1080 p recording at 30 fps, and taking 14 MP (4384 × 3288 px) photos. However, certain platforms support the use of other cameras than the proprietary ones. Furthermore, DJI specifies an extended range of operation for the newer products, up to 2 km.

In contrast with the above-listed RTF vehicles, which are readily assembled UAVs with the possibility of limited customization, 3D Robotics and the Arducopter come with modular solutions, based on the Arduino platform [90]. These UAVs use Ardupilot Mega (APM) or Pixhawk flight controllers that are known to be custom-programmable. Furthermore, these controllers support the attachment of various external devices, such as sensors, controllers and communication units. The Arducopter UAVs are usually custom-built, but there exist complete kits, as well, offering RTF or almost-RTF solutions. An RTF quadrotor is the 3DR Iris+, which costs $750 and weighs 1300 g [91], whereas an almost-RTF solution is the Arducopter Quad [6], costing $620 and having a similar weight. Compared to the platforms from the other producers, the Arducopter UAVs are highly customizable, though requiring more knowledge of UAV programming and operation. A more recent platform is the 3DR Solo, a user-friendly RTF UAV [92] with enhanced capabilities (among others, increased flight time up to 20 min with a mounted GoPro camera and an improved onboard controller).

Regarding sensors in Arducopters, these platforms come with the ability of customization. The newer Pixhawk controller comes with built-in gyroscopes, accelerometers and pressure sensors [93]. On the vision part, the Iris+ has, for instance, the possibility of mounting an external camera using a gimbal system. Pixhawk offers an optical flow module called PX4FLOW[94], which can be used for navigation purposes. It is not meant for video recording, though, due to its reduced performance (it has a resolution of 752 × 480 px).

The presented UAVs are meant mainly for aerial imaging and gaming applications. Despite the different price ranges, the types of sensors used for stabilization are similar. Obviously, the more costly solutions offer better stabilization. On the vision side, platforms supporting the mount of external cameras offer improved recording experience. However, working with integrated vision units eases the usage of the platform, as in the case of the AR.Drone or with the PX4FLOW module. The quality and frame rate offered, for example, by the AR.Drone 2.0 are already good enough for use for vision-based navigation and environment capturing.

## 5. UAVs for Infrastructure Inspection Applications

A growing interest is shown for using UAVs for inspection and other remote sensing applications, starting from public area monitoring, to infrastructure inspection, intelligent farming or aerial mapping. Reaching satisfactory performance with low-cost UAVs can offer new perspectives for industrial applications and public services.

As we discuss in the sequel, several projects already focus on inspection use-cases, although they use more costly UAVs. We present specific applications by grouping them into two common classes, namely power line inspection and building monitoring. We also dedicate a subsection to railway inspection applications, a less explored field. Furthermore, we highlight the level of autonomy of the UAVs used in the works discussed. Finally, we list and discuss briefly the sensors used in the presented applications.

### 5.1. Power Line Inspection

Power line inspection applications roughly cover the tasks of following the lines and stopping at interesting points, to scan in detail certain parts of the infrastructure. This procedure is similar to the case of inspecting pipelines or any other linear structures, such as roads, walls, coasts, *etc*. Although projects can be found that target gas pipeline monitoring [95] or structure inspection in general [96], the topic of power line inspection appears to be more popular in recent years.

In their survey in 2010, Montambault *et al.* [97] already point out several research projects all around the world that use quadrotors for power line inspection. As stated in the dissertation of Ellis, in August 2013, in Australia alone, there were already 16 companies listed as licensed UAV operators for power line inspection [98]. We do not have details on the level of automation in these applications. However, the following projects clearly focus on automating power line inspection.

Li *et al.* [4] already perform fully-automated power line inspection. They use a helicopter of 31 kg, which also carries enough fuel for up to one hour of flight. Furthermore, their helicopter is equipped with a more advanced sensor suite, which eases flight automation. Although promising, such platforms do not fit into the inexpensive category.

Several projects strongly focus on the image processing part of inspection, from various perspectives. Zhang *et al.* [99], for instance, compare some line detection algorithms for identifying and tracking power lines from videos captured by quadrotors. Luque-Vega *et al.* [100] combine a color camera with a thermal infrared camera to inspect infrastructure components. Larrauri *et al.* [101] deal with calculating distance to vegetation, trees and buildings, based on video frames. Martinez *et al.* [102] perform power line tower inspection. Their tracking approach steers the camera so as to keep an inspected tower in focus while flying along lines.

### 5.2. Building Monitoring

Another popular application field is the inspection of building façades and other surfaces, with the aim of examining their integrity. In such use-cases, the primary goal is to design flight plans that allow for proper data acquisition.

Baiocchi *et al.* [3] use quadrotors in a post-seismic environment for inspecting historic buildings for cracks and other damages. The GPS-based path planner developed by them optimizes flight paths, reducing redundant acquisition. Furthermore, their processing software allows for the 3D reconstruction of building façades from pairs of images. Another project, led by Eschmann *et al.* [103], presents a similar application, though with manual flight control.

Nikolic *et al.* [104] deal with power plant boiler inspection. They design an automated trajectory-following system and a personalized sensor suite for visual navigation. Using these, they examine the interior walls of boilers, expanding the GPS navigation functionality of the quadrotor with visual navigation, in order to be able to operate in GPS-denied regions, as well.

### 5.3. Railway Infrastructure Inspection

Railway inspection comprises the tasks of structure inspection and linear structure following. In this context, railway inspection is related to the previous two fields. It is an application area not yet considered in public research projects, to the best of our knowledge. As presented below, newsletters report on several companies that intend to or already use UAVs in railways, based mainly on manual teleoperation or automated waypoint navigation. However, we have limited technical information on the work performed by these groups.

In the spring of 2013, news appeared about German Railways (DB – Deutsche Bahn) regarding their intention of using UAVs for catching graffiti sprayers [105]. French National Railways (SNCF – Société nationale des chemins de fer français) announced in the autumn of 2013 a project of railway bridge and viaduct inspection using UAVs [106]. The international company Elimco is also offering inspection of various infrastructures, including railways [107]. Similar applications can be found at Microdrones, where automated waypoint navigation is already implemented [108]. An article from Smartrail World reports on further companies that use or plan to work with UAVs: NetworkRail from the UK, ProRail from The Netherlands, Union Pacific from the USA and the Jerusalem light rail network [109]. Although few technical details are publicly available about these projects, they mainly seem to be based on manual teleoperation.

### 5.4. Sensors in Infrastructure Inspection Applications

Most papers discussed above provide generic or little information about the sensors used. In general, some kind of color cameras are used to perform data acquisition. From the works where more details are provided, Hausamann *et al.* [95] use a combination of optical and infrared sensors, discussing sensor types with different spectral bands; and synthetic aperture radars that have higher availability than color cameras due to the fact that radars are independent of weather and light conditions. Luque-Vega *et al.* [100] combine thermal-infrared cameras with color cameras for improving background subtraction and, thus, object detection. Larrauri *et al.* [101] use an HD camera in their project, though for processing data at only 4 fps. Eschmann *et al.* [103] perform navigation based on GPS data and use an external 12-MP commercial camera for offline post-processing only. Nikolic *et al.* [104] consider a CMOS image sensor and low-cost inertial sensors on a custom-built integrated circuit that aids UAV navigation and data collection in GPS-denied environments.

From these various details, one can conclude that color cameras are usually considered for data acquisition. Often, the performance of these cameras is reduced, but still good enough, while aiming at keeping the device low cost or at respecting payload constraints. Among the applications discussed, in certain cases, infrared cameras are additionally used to improve detection.

## 6. Illustration in Railway Inspection Use-Cases

Finally, we illustrate in this section the use of the camera-based sensing and control methods presented in Sections 2 and 3 for automated railway inspection. We formulate two use-cases: one where the UAV performs infrastructure inspection in close, but difficult-to-access areas (such as long bridges or tracks separated from the road by, e.g., a river). A second use-case is meant for railway track following for the sake of recording the infrastructure, such as tracks, sleepers, points or cabling. In the first use-case, the automated control runs on a remote station, while in the second one, full control is onboard. The chosen UAV platform is the Parrot AR.Drone 2.0 quadrotor, presented in Section 4. In certain cases, the implementation ideas come from the specifics of this particular platform, but they are usually valid for other low-cost quadrotors, as well.

We continue with evaluating the vision methods presented in Section 2, namely edge detectors for rail track detection in Section 6.1 and feature detectors for target (signal light) detection in Section 6.2. Then, we break down the use-cases into subtasks. We select detection solutions based on the results from our experiments detailed in Sections 6.1 and 6.2. Furthermore, we indicate other vision and control methods needed for our setup, based on the discussions from Sections 2 and 3. In this manner, our method selection from Section 6.3 considers, to an extent, our own experimental results and further solutions from the conclusions of our survey.

### 6.1. Evaluating Feature Detectors for Target Detection

In the first use-case, the quadrotor must find the target object before inspecting it. Considering that the target is close enough to the quadrotor (below 10 m) and is in its field of view, one can apply feature detection, description and matching methods that, given a reference image of the target, can identify it in a new image, called the scene image.

Numerous detection, description and matching methods are readily implemented. We consider the OpenCV 2.4.9 library [110] that, from the methods discussed in Section 2.1, comes with implementations for the SURF, SIFT, FAST and Shi–Tomasi feature detectors and the SURF, SIFT, BRIEF and ORB descriptors. Each of these methods has a number of tuning parameters, as presented on the OpenCV website [110]. Based on prior tuning tests, we select a grid of meaningful values for these parameters (values marked with bold are the best according to the optimization criterion we describe below).

– For SURF, the Hessian threshold is taken in {300, 310, …, **420**}, the number of octaves in {**2**, 3, …, 8} and the number of octave layers in {1, 2, 3, **4**, 5, 6}, and we will use up-right features and extended descriptors.
– For SIFT, we allow all features to be retained, and we set the number of octaves in {1, **2**, 3}, the contrast threshold in {0, 01, **0.02**, …, 0.16}, the edge threshold in {5, **7**, …, 15} and the standard deviation of the Gaussian in {1.2, 1.3, **1.4**, …, 2:1}.
– For FAST, we use threshold values between {0, 1, …, **31**, …, 80} and test both **enabling** and disabling non-max suppression.
– For Shi–Tomasi, we allow all features to be retained, set a quality level from {0*.*001, 0*.*006, …, **0.021**, …, 0*:*041}, a minimum feature distance of {0, **1**, …, 5}, an evaluation block size of {3, 4, 5, **6**}, allow for enabling or **disabling** the Harris detector and set parameter *k* to {0*.*02, **0.04**, …, 0*.*1}.

With each setting, we combine the detectors with all of the descriptors and a FLANN-based matcher [38] in order to evaluate the performance of the methods. The descriptors and the matcher require no parameter tuning.

**Figure 3:**
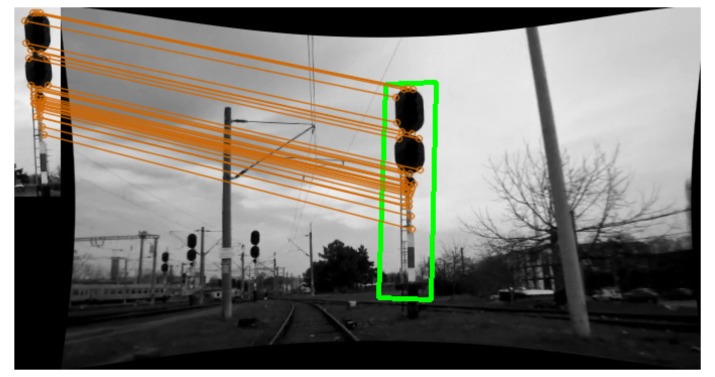
Target detection. Matching result with the reference and scene image.

As the data source, we consider a 46×188 px reference image and 50 scene images, all 640×360 px in size and rectified. The reference image was taken with the same camera as the scene images. Each scene image contains the target object at different distances. Figure 3 shows a sample matching result.

After testing the various combinations of detector and descriptor methods, we obtain meaningful results only when using the SIFT descriptor. The other descriptors, in general, fail to detect the target object. Then, testing all of the detectors on the grid of parameters, we select the best parameter set for each detection algorithm that maximizes the detection rates during the simulations. Table 1 summarizes the performance of the four detectors in the case of these parameter sets.

As shown in Table 1, we evaluate the detection success rate, the average execution time per frame and average errors on the horizontal position and the scaling of the detected object. The detection rate tells in how many frames the algorithm detected the reference object. The average execution time considers the total time required for detection, description and matching, for a single frame, *i.e.*, the total image processing time. The average position error tells the horizontal distance in pixels between the real center of the object in the scene image and the center of the detected area. Finally, the scaling error indicates the average difference between the size of the reference image and the size of the detected object, expressed in percentages, *i.e.*, it is a size detection error. Note that the latter two parameters indicate the accuracy of the detection, while the first two inform about the usefulness of the algorithm in the case of online use.

According to the results from Table 1, the FAST and Shi–Tomasi methods outperform the other two, both in detection rate and execution time. The detection rate of SIFT is also acceptable. Looking at the average position error, all of the methods perform well, except SURF. However, the scaling error indicates the opposite. SURF, although it has a far lower detection rate, detects the object size more precisely. Furthermore, higher scaling errors in the case of the other methods appear since the object detection algorithm was implicitly allowed to have these errors in favor of higher detection rates by the optimization procedure. Additionally, the small position errors indicate that, with the last three methods, the target was identified in the correct place.

Based on these results, we select the FAST algorithm for target detection in the given use-case, in combination with the SIFT descriptor. However, we remark that the parameter sets were fine-tuned for the given dataset, and other combinations might turn out to have better results for different scenarios where other reference/target objects or other image parameters are considered. Nevertheless, we highlight the good performance of the FAST and Shi–Tomasi detectors, which, with an average execution time below 10 ms, allow for at least 100-Hz control, good enough for target detection during flight with a quadrotor. With respect to the discussion in Section 2.1, the selection of the Shi–Tomasi detector confirms the conclusions from there. However, the results of the FAST detector are even better in our particular setup, while the Harris detector seems to reduce the performance when applied in the Shi–Tomasi algorithm.

### 6.2. Evaluating Edge Detectors for Track Detection

In the second use-case, the quadrotor has to follow track lines. This can be accomplished by vanishing point-based control, as introduced briefly below in Section 6.3.1 and presented in [111]. To achieve this aim, edge detector methods can be used in combination with line extractors that help with finding the track lines, which are then used to find their vanishing point.

**Figure 4:**
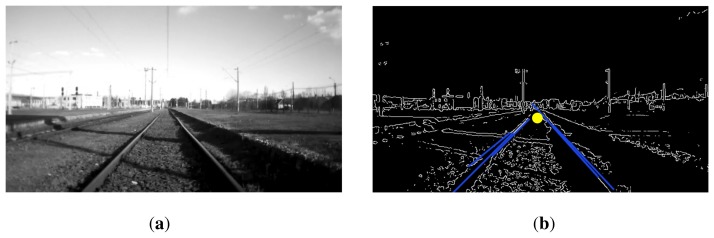
(**a**) Track line detection; (**b**) Canny edge detection result and vanishing point detection.

From the edge detectors mentioned in Section 2.1, OpenCV comes with implementations for Canny, Sobel, Laplacian and Scharr algorithms. We will test these methods on a grid of meaningful parameter sets and combine them with a probabilistic Hough transform (PHT) for line extraction and a custom filtering method for line selection. This method removes all of the lines up to a vertical angle, after which it progressively filters out the lines that do not point to an average vanishing point. An example of the processed image and obtained lines and vanishing point is shown in Figure 4.

The parameters of the detectors are taken according to the following grid (best values marked with bold or stated separately).

– For all of the methods, we worked with default image color depth and edge modeling border type and set the *x* and *y* derivative orders in {0, 1, 2}, kernel sizes in {1, 3, 5, 7}, derivative scaling factors in {0.001, 0.002, …; 0.01, 0.015, …, 0.14} and a delta value, added to the pixels of the output image during the convolution, in {0, 0.01, …, 0.40}. Note that some parameters appear with only some of the methods.
– In the case of Canny, the two hysteresis thresholds are taken in the interval {0, 10, …, 90, 91, …, **106**, …, 110, 120, …; 300g; the best kernel size was 3, and we allowed for **enabling** or disabling the use of the *L*_2_ gradient in image gradient magnitude calculation.
– For Sobel, the best values of the parameters are: derivative orders *x* = 1 and *y* = 0, kernel size three, scaling factor 0.0125 and delta 0.037.
– For Laplacian, the best values are: kernel size five, scaling factor 0.002 and delta 0.095.
– For Scharr, the best values are: derivative orders *x* = 1 and *y* = 0, scaling factor 0.001 and delta 0.23.

From the common parameters, the kernel size is the size of the matrix used in calculating the transformed image. In our experiments, we observe that keeping this value low provides better results. The scaling factor determines the image dimming, and better results are obtained when keeping this value low, *i.e.*, having almost completely dimmed images. The delta value has no visible influence on the image processing, although it turns out that the lower its value, the better the detection.

We applied these detectors for a set of 165 scene images of size 640 × 360 px, all containing a pair of track lines with different orientations, after which the line extraction and selection algorithms were executed. We evaluated the average execution time and the average and maximum position errors. The execution time is calculated per frame, for the detection algorithms only, as these are the subject of our evaluation. The position errors determine the difference in pixels between the real (ground truth) and measured horizontal position of the vanishing point of the tracks. Based on these indicators, Table 2 summarizes the performance of the detection methods for the parameter sets for which the average position error was the lowest.

From Table 2, one can see that all of the methods have an execution time below 3 ms. Recall that we considered only the duration of the detection, which together with the line extraction and selection results in times up to 20–25 ms. Still, this offers a 40–50-Hz control rate in the case of any detection algorithm, high enough for quadrotor control. The maximum position error indicates some false detections, which is the least severe in the case of the Laplacian algorithm. However, given the 640 px image width, all of the methods have an average position error below 2.5% that indicates an overall correct detection. From all of these parameters, we prefer to consider the position error the most important and use, therefore, the Laplacian method in our use-case of track following. Nevertheless, the test results confirm the discussion from Section 2.1 on the good performance of the Canny detector and its weakness of generating false positives, when comparing with the performance of the Laplacian method.

### 6.3. Use-Case Subtasks

We select solutions for the two use-cases, the short-range inspection in difficult-to-access areas and the long-range, track following-based infrastructure recording. This selection is just an illustration of how the presented methods can be used for UAV navigation. We provide no details on the settings of the considered techniques. However, flight tests were also already performed that demonstrate the suitability of several selected methods.

First, we need to break down the use-cases into subtasks. These are mainly: take-off, fly on a path, find and inspect targets, fly home and land. Next, we detail these subtasks and propose solutions to the related control problems. The take-off and landing tasks are, in the case of the AR.Drone and with many other RTF quadrotors, already solved by built-in functions. Furthermore, the newest products come with a fly-home function that, based on GPS coordinates, makes the quadrotor return autonomously to its take-off location and land there. For automation of the other tasks, additional processing and control methods are required.

#### 6.3.1. Flying on a Path

Flying on a path poses different challenges in the two use-cases. In the case of remotely-controlled local inspection, it can be solved by GPS waypoint navigation with obstacle avoidance. Here, three subtasks can be identified: planning the waypoint sequence, navigating using GPS data and obstacle detection and avoidance. The waypoints have to be planned, e.g., to optimize the flight time or to avoid obstacles. For online planning, we suggest the use of the RRT and MPC-based algorithms. Regarding GPS-based waypoint navigation, quadrotors with a GPS module like the AR.Drone usually have implementations for this task. We will consider the software from [112]. Finally, obstacle avoidance is one of the most challenging tasks for quadrotors. Here, based on the experiments from Section 6.1, we suggest using Laplacian filtering for detection, combined with optical flow techniques that can determine motion. Then, we recommend the previously mentioned online planning methods for the avoidance maneuver.

In the second use-case, railway following, the path planning and following problem boils down to line following. With an onboard camera looking ahead, this can be achieved, for example, by finding and tracking the vanishing point of the lines formed by the pair of rails [111]. The vanishing point detection consists of image preprocessing for edge detection, line detection and line filtering in order to identify the tracks and their vanishing point. Based on [111] and on our experiments from Section 6.2, we propose the use of the Laplacian operator for edge detection. Then, the probabilistic Hough transform (PHT) can be applied to find lines from the resulting contours. These lines can then be filtered simply based on their lengths and slopes: we select long enough lines (e.g., longer than a quarter of the image height) that are almost vertical. These lines have a vanishing point that matches the vanishing point of the tracks. The tracking subtask can be solved by simple visual servoing: the vanishing point can be kept in the middle of the camera image through a proportional derivative controller. More precisely, we propose to perform yaw (*z* axis) angle rotations, while additionally correcting with lateral displacement and forward velocity reduction if the vanishing point is outside the desired range.

#### 6.3.2. Finding and Inspecting Targets

For both use-cases, a method to find the target and a navigation strategy are needed, whereas in the first use-case (local inspection), a further navigation solution is needed for inspection. We consider a database of images of the possible targets. Then, based on the conclusions from Section 6.1, we propose to use FAST feature detectors with SIFT descriptors and the FLANN-based matcher. Together, these methods can track an object in subsequent frames. Above a threshold for the detection rate over time, we consider the target being found. We also point out the solution presented in [102], where machine learning is used in combination with edge detectors to find the target using a database of reference images. Yet another idea is to test RANSAC model fitting, introduced in Section 2.1.

To navigate around a detected target, we consider visual servoing techniques. An alternative would be GPS-based navigation, although for the AR.Drone, the GPS accuracy is of 2m [113], not enough for safe navigation close to objects. We propose therefore the use of PBVS methods, introduced in Section 2.3. Furthermore, based on our experiments from Section 6.1, a basic visual servoing solution is to use the target detection described above together with homography-based identification. The obtained homography can then be used to determine the scaling and image-frame position of the target. Knowing the distance to the target in the reference image, the scaling and image-frame position can be then transformed into longitudinal and lateral distances to the object, which indicate the relative position to the target. Based on this, simple controllers can be applied to correct the distances so as to track the desired inspection trajectories.

## 7. Summary and Outlook

In the first major part of this paper, we reviewed vision and control methods for UAVs, with the final goal of using them in railway inspection applications. In the second part, we presented several popular low-cost quadrotor platforms, overviewed research concerning UAV inspection applications and formulated two use-cases. The use-cases address the novel application field of railway inspection and focus on short-range inspection in difficult-to-access areas and long-range track following. We performed an exhaustive evaluation of feature detectors for track following and target detection. Finally, we devised a strategy to accomplish the use-case task using results from our experiments and from the conclusions of the survey.

The survey of vision and control techniques revealed several open challenges, like the difficult problem of fine-tuning in the case of vision methods, the high computational demands of both vision and flight planning tools compared to the onboard processing capacity of the low-cost UAVs or the limitations appearing due to the lack of adequate mission formalization and due to the restrictive regulations. Further open issues are the lack of a general obstacle avoidance solution, which is crucial for fully-automated UAV navigation, and limitations derived from the short battery life of low-cost UAVs. Our future work is motivated by the railway inspection use-cases, and we are currently continuing our research by evaluating additional vision techniques for object classification and for obstacle avoidance, by developing trajectory and path planning techniques for automated flight of low-cost quadrotors.

## Figures and Tables

**Table 1:** Performance evaluation of the feature detectors for target detection.

**Method**	**Detect. Rate (%)**	**Execution Time (ms)**	**Position Error (px)**	**Scaling Error (%)**
SURF	8	71.5	41.7	5
SIFT	54	17.4	2.7	45
FAST	98	8.3	2.2	37
Shi-Tomasi	96	7.4	2.2	41

**Table 2:** Performance evaluation of edge detectors for line detection.

**Method**	**Exec Time (ms)**	**Average Position Error (px)**	**Max Positioning Error (px)**
Canny	1.60	17.5	126
Sobel	2.27	16.4	193
Laplacian	2.74	13.8	79
Scharr	2.19	16.9	193
